# Host Genetic Impact on Infectious Diseases among Different Ethnic Groups

**DOI:** 10.1002/ggn2.202300181

**Published:** 2023-11-05

**Authors:** Lisa Naidoo, Thilona Arumugam, Veron Ramsuran

**Affiliations:** ^1^ School of Laboratory Medicine and Medical Sciences College of Health Sciences University of KwaZulu‐Natal Durban 4041 South Africa; ^2^ Centre for the AIDS Programme of Research in South Africa (CAPRISA) University of KwaZulu‐Natal Durban 4041 South Africa

**Keywords:** COVID‐19, human immunodeficiency virus, host genetics, malaria, tuberculosis

## Abstract

Infectious diseases such as malaria, tuberculosis (TB), human immunodeficiency virus (HIV), and the coronavirus disease of 2019 (COVID‐19) are problematic globally, with high prevalence particularly in Africa, attributing to most of the death rates. There have been immense efforts toward developing effective preventative and therapeutic strategies for these pathogens globally, however, some remain uncured. Disease susceptibility and progression for malaria, TB, HIV, and COVID‐19 vary among individuals and are attributed to precautionary measures, environment, host, and pathogen genetics. While studying individuals with similar attributes, it is suggested that host genetics contributes to most of an individual's susceptibility to disease. Several host genes are identified to associate with these pathogens. Interestingly, many of these genes and polymorphisms are common across diseases. This paper analyzes genes and genetic variations within host genes associated with HIV, TB, malaria, and COVID‐19 among different ethnic groups. The differences in host–pathogen interaction among these groups, particularly of Caucasian and African descent, and which gene polymorphisms are prevalent in an African population that possesses protection or risk to disease are reviewed. The information in this review could potentially help develop personalized treatment that could effectively combat the high disease burden in Africa.

## Graphical abstract

Despite efforts in developing treatments, malaria, tuberculosis (TB), human immunodeficiency virus (HIV), and coronavirus disease of 2019 (COVID‐19) remain a global health burden, especially in Africa. Disease susceptibility and severity varies among individuals. Host genetics potentially contribute a significant portion to disease susceptibility and severity. Common host genes and polymorphisms that associate with these pathogens are reviewed here.

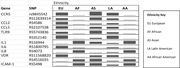

## 1 Introduction

In 2019, the top ten causes of death were due to non‐communicable diseases (NCDs) worldwide. The most common NCDs such as cardiovascular disease, diabetes, chronic respiratory disease, and cancer make up 55% of deaths worldwide.^[^ ^1^, ^2^ ^]^ All NCDs are responsible for 41 million deaths a year, ≈74% of all deaths.^[^ ^3^ ^]^ However, in 2019, in Africa, the three infectious/communicable diseases: tuberculosis (TB), malaria, and human immunodeficiency (HIV) were the cause of the top 8 most common causes of death.^[^ ^4^, ^5^, ^6^, ^7^ ^]^ In other continents of the world, communicable diseases are rarely among the top ten causes of death. In addition, the recent pandemic caused by severe acute respiratory syndrome coronavirus 2 (SARS‐CoV‐2) has drastically impacted global death rates over the past few years. Europe had the highest corona disease of 2019 (COVID‐19) cases of 273 666 626, followed by the Western Pacific and America. However, Africa had the lowest number of COVID‐19 cases of 9 500 642. On November 18, 2022, there were 257 984 COVID‐19 deaths in Africa.^[^ ^8^ ^]^ In Europe, on January 13, 2023, there were 2 169 191 COVID‐19 deaths.^[^ ^9^ ^]^ In 2019, the death rate in Africa was 7.77 per 1000 people. In 2020, when the first case of COVID‐19 was identified, the death rate reduced slightly to 7.62 deaths per 1000.^[^ ^10^ ^]^ In Europe, the death rate was 9.97 in 2019 and increased to 11.1 in 2020.^[^ ^10^ ^]^ This was also observed in North and South America.^[^ ^10^ ^]^ Despite Africa's COVID‐19 data being underrepresented due to lower testing capacity,^[^ ^11^ ^]^ we noticed that the death rate reduced after COVID‐19. This indicates that COVID‐19 had a less severe impact on the African population as opposed to continents with a predominantly Caucasian population.

High levels of infectious disease cases in Africa have been attributed mainly to poor socioeconomic status resulting in a lack of basic needs and poor living conditions.^[^ ^12^ ^]^ Another contributing factor to the higher infection rates in Africa could be due to an ethnic‐specific bias as the African population consists of 980 million black individuals.^[^ ^13^ ^]^ Previous studies showed that different populations react differently to infectious diseases based on their genetics.^[^ ^14^, ^15^, ^16^ ^]^ Africa, being the second largest continent with the second largest population, is known to have the greatest genetic diversity with 3000 different ethnic groups populating the continent. The high level of genetic diversity is a challenge and a prospect for Africa, as it is the least studied genome with one of the highest health burdens.^[^ ^17^ ^]^ Despite immense efforts toward developing effective preventative and therapeutic strategies for these pathogens, several uncured diseases with unsafe and resistant drugs are on the market. New treatment strategies that are affordable, harmless, and effective for Africans need to be developed for these infectious diseases. In this review, we determine the host genetic contributions to these diseases in the African population and whether the African population's genetics contribute to high rates of infectious diseases. The host genetics associated with these diseases need to be studied more in an African population.

Several host genes are associated with numerous infectious diseases, one of them being the vitamin D receptor (*VDR*). The *VDR* gene is a transcription factor that controls the expression of various genes involved in physiological processes,^[^ ^18^ ^]^ such as immunity and cell differentiation to target tissues.^[^ ^19^ ^]^ *VDR* was suggested to be important in malaria, TB, HIV, and SARS‐Cov‐2 susceptibility and COVID‐19 severity in different populations.^[^ ^14^, ^20^, ^21^, ^22^ ^]^ Polymorphisms within host genes, can alter the gene function and lead to anti or pro‐disease activity.^[^ ^23^, ^24^, ^25^, ^26^ ^]^ Several common host genes and gene polymorphisms play a role in infectious diseases.

Unraveling the host–virus and virus–virus interaction and identifying host factors that are important for pathogenesis will better equip us to understand the variability in pathogenesis among different ethnic groups, particularly between Africans and Caucasians, and develop personalized treatments for these highly infectious diseases in Africa. This review will examine host genes overlapping with HIV, TB, malaria, and COVID‐19. We will analyze the host gene functionality, genetic variations if any, and their effect, or whether it might confer protection or risk to infected individuals from different ethnicities and allele frequency among different ethnic groups.

## 2 Inclusion and Exclusion Criteria for Selected Genes and SNPs

**Figure 1:**
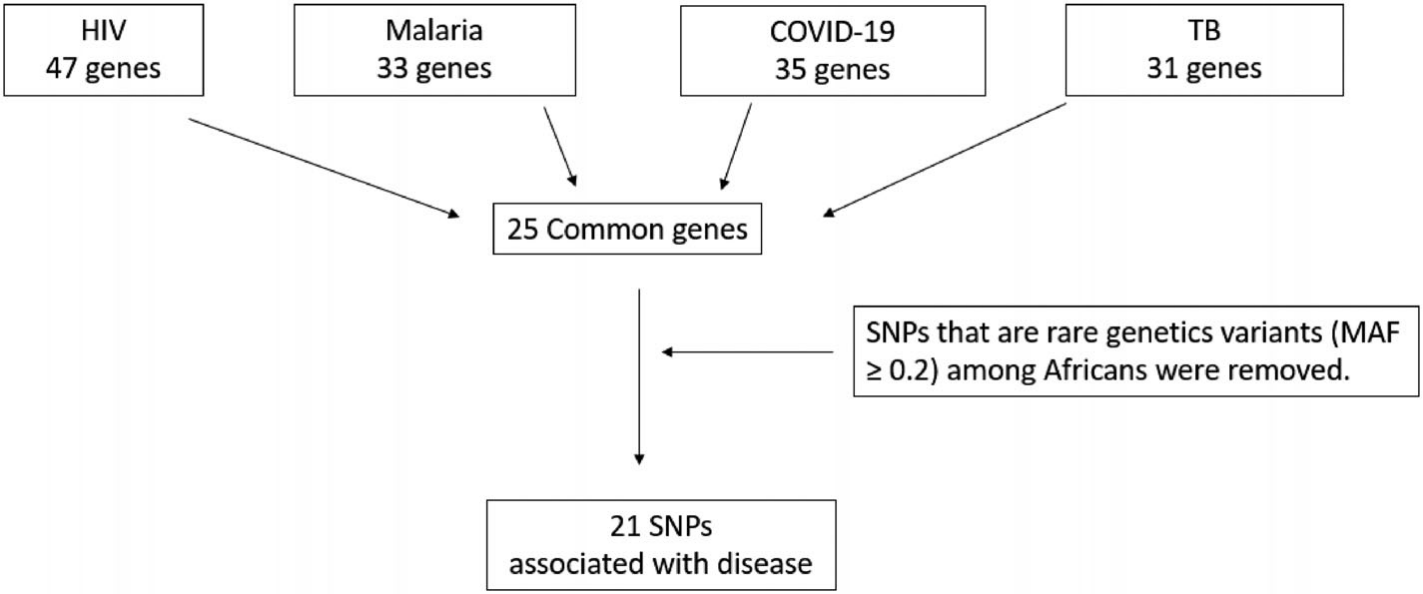
Flow diagram of the inclusion and exclusion criteria of genes and SNPs discussed in this review.

Host genes associated with HIV were identified using Google Scholar using the search words “host genes associated with HIV susceptibility, control, and progression” between 2007–2023 and sorted by relevance in the first phase. There were 19 900 results on Google Scholar on June 3, 2023. We looked at all the titles in the second phase and found 25 relevant articles. In the third phase, we read all abstracts and selected 6 relevant articles. HIV‐associated host genes from these relevant articles selected were used as a basis for the selection of host genes associated with TB, Malaria, and COVID‐19 in phase four. The articles for these genes were found on PubMed using the infectious disease and the gene name. SNPs within the host genes that were associated with these infectious diseases were identified by these published scientific papers. There were multiple SNPs for each gene (*VDR*, *n* = 6; *MBL*, *n* = 4; *ICAM‐1*, *n* = 3, *CXCR6*, *n* = 1, *CX3CR1, n* = 0*, CCR2, n* = 1*, CCR5, n = 4, CXCR4, n* = 0*, SDF‐1, n* = 0*, IFN, n* = 0*, TNF, n* = 0*, CCL3*, *n* = 1, *CCL5*, *n* = 1, *CCL2, n* = 4, *IL‐1*, *n* = 7, *IL‐4*, *n* = 1, *IL‐6, n* = 10, *IL‐8*, *n* = 1, *IL‐10, n* = 3*, IL‐18*, *n* = 0, *TLR2*, *n* = 0, *TLR4, n* = 4, *TLR7*, *n* = 0, *TLR8*, *n* = 0, *TLR9*, *n* = 6). The resulting SNPs were then ranked in order of minor allele frequencies (MAF) based on data from the National Centre for Biotechnology Information (NCBI dbSNP) (https://www.ncbi.nlm.nih.gov/) and SNPs characterized as being rare genetic variants among the African population (MAF < 2 or 1%) were removed (*VDR*, *n* = 2; *MBL*, *n* = 2; *ICAM‐1*, *n* = 2, *CXCR6*, *n* = 1, *CX3CR1, n* = 0*, CCR2, n* = 0*, CCR5, n = 2 CXCR4, n* = 0*, SDF‐1, n* = 0*, IFN, n* = 0*, TNF, n* = 0*, CCL3*, *n* = 0, *CCL5*, *n* = 1, *CCL2*, *n* = 1 *IL‐1*, *n* = 1, *IL‐4*, *n* = 1, *IL‐6*, *n* = 2 *IL‐8*, *n* = 1, *IL‐10*, *n* = 3, *IL‐18*, *n* = 0, *TLR2*, *n* = 0, *TLR4*, *n* = 0, *TLR7*, *n* = 0, *TLR8*, *n* = 0, *TLR9*, *n* = 2). MAF, disease associations, and potential mechanisms of action for all eligible genes and SNPs were then summarized in **Figure** **1**. Information regarding the gene function, genetic variation, and effect on disease has been summarized in **Table** **1**.

**Table 1:** Genes associated with infectious diseases

Gene name	Function	HIV	Malaria	COVID‐19	TB	Genetic variation (>0.2)	Reference
Chemokine receptors							
*CCR2*	Involved with the innate immune response.	Co‐receptor of HIV, alters the cell surface location of the *CCR5* and *CXCR4* and regulates heterodimerization, reduces levels of *CXCR4* in PBMCs from healthy donors and late disease.	*CCR2*‐deficient mice have persistent parasitemia in malaria. *CCR2* deficient mice inhibit inflammatory monocyte recruitment to the site of infection.	*CCR2* is associated with the monocyte recruitment and infiltration of these cells in the lungs of COVID‐19‐infected individuals. CCR2 may be protective in SARS‐CoV‐infected dendritic cells. *CCR2* reduced viral load in SARS‐CoV‐2 infected mice.	*CCR2* contributes to the defense against MTB. This was shown in a study with *CCR2*‐deficient mice.		[27, 28, 29, 30, 31, 32, 33, 34]
*CCR5*	Associated with cell activation and migration.	HIV receptor for HIV entry.	*CCR5* was associated with adverse effects caused during pregnancy with malaria infection. *CCR5* deficiency increases maternal parasitemia.	*CCR5* is expressed on macrophages and T cells acting as a co‐receptor for SARS‐Cov‐2. *CCR5* plays a role in COVID‐19 severity.	*CCR5* plays an essential role in cell activation and migration in immune responses against TB.	rs9845542 severe COVID‐19	[26, 35–49]
						rs12639314 severe COVID‐19	
*CXCR4*	Regulates and expresses T cell migration	*CXCR4* s play a role as co‐receptors for HIV, it is important at a late stage of disease development.	*CXCR4* increases calcium levels which influence sporozoite transformation into exoerythrocytic forms which is necessary for parasite progression in the liver. Eliminating *CXCR4* inhibits the development of *Plasmodium falciparum* in the liver.	High levels of *CXCR4* direct cells to destroy lung tissue.^[^ ^50^ ^]^ Fatal COVID‐19 is associated with elevated *CXCR4* in bystander T cells.	*CXCR4* expression is higher among TB/HIV co‐infected individuals than HIV infected individuals Increased *CXCR4* expression is associated with TB in alveolar macrophages.		[50, 51, 52, 53, 54]
*CX3CR1*	Involved with adhesion and migration.	Co‐receptor for HIV infection, HIV‐1 immune cell recruitment, infection expansion, signaling, and regulation of cell function.	High *CX3CR1* and *CCR2* on CD14 monocytes limit parasitic growth through antibody dependent cellular inhibition activity. Similarly, in another study, high *CX3CR1* expressing cells were associated with better survival.	*CX3CR1* expression was impaired among individuals who had contrary artery disease and SARS‐CoV2.	The *CX3CR1* expression levels on monocytes are elevated among latent TB infections.		[25, 55–59]
*CXCL12*	Involved in a wide range of functions, one being the ability to control the trafficking of leukocytes.	Ligand for *CXCR4*, which induces receptor internalization, and might restrict transmission of X4 viruses.	High *SDF‐1* levels in African children were associated with acute malaria compared to healthy children.	*SDF‐1* is upregulated early post‐SARS‐CoV‐2 infection.	*SDF‐1* is a diagnostic marker for TB and correlates with severe TB.		[60, 61, 62]
*CCL2*	Pro‐inflammatory chemokine.	Granulomatous reaction in lung tissue.	High levels of *CCL2* were observed in *P. vivax* infections.^[^ ^63^ ^]^ Similarly, elevated *CCL2* has been found in malaria‐infected placentae.^[^ ^64^ ^]^	The *CCL2* chemokine is involved in the migration of inflammatory monocytes. It is produced by alveolar macrophages, T cells, and endothelial cells. In addition, *CCL2* chemokines were expressed at higher levels in lung macrophages of severe COVID‐19 patients. *CCL2* was upregulated early post‐SARS‐CoV‐2 infection.	*CCL2* is associated with TB.	rs4586, resulting in a T>C allele change, affecting TB susceptibility.	[31, 63–69]
*CCL3*	Involved in the recruitment of neutrophils and monocytes, and the activation of inflammatory signaling.	Co‐receptor of *CCR5*, internalizing and lessening the *CCR5* levels on the cell surface, cognate ligand for *CCR6*, natural ligands of *CCR5*. blocks HIV‐1 infection.	*CCL3L1* CNV and its association with disease susceptibility.	The *CCL3* levels were increased in CM‐infected mice.^[^ ^70^ ^]^ In addition, elevated levels of monocyte‐attracting *β*‐chemokines CCL3 have been found in malaria‐infected placentae.	*CCL3* is a leucocyte activating chemokine that is involved in TB restriction. High levels of *CCL3* were detected in PTB when compared to latent TB (LTB) and healthy control (HC) patients.		[16, 64, 70–83]
*CCL5*	Involved with the migration of macrophages and NK cells.	Natural ligand of *CCR5*, blocks HIV infection, reduces CD4 T cell depletion.	Malaria outcomes are influenced by the role of *RANTES* in host immunity. Low levels of *RANTES* were associated with CM in Ugandan children. Bujarbaruah et al. observed similar results, showing low levels of *RANTES* were associated with severe malaria and high *RANTES* was associated with malaria recovery and uncomplicated malaria.	*CCL5* is upregulated in early post‐SARS‐CoV‐2 infection. In addition, high levels of *CCL5* in found in critically ill COVID‐19 patients.	*CCL5* plays a key role in co‐stimulation of T cell proliferation and *RANTES* activation in anti‐mycobacterial immunity. Studies have shown a significant relationship between *RANTES* polymorphisms and an increased TB risk.	(rs2107538) C>T increased PTB susceptibility.	[84, 85, 86, 87, 88, 89, 90, 91]
Toll‐like receptors							
*TLR2*	*TLR* identifies motifs in pathogens.	Causing HIV‐1 expression during opportunistic co‐infections.	*TLR2* helps the recognition of a wide range of ligands. Sporozoite activates *TLR2* and induces macrophages to release proinflammatory cytokines.	*TLR2 is* theoretically important in COVID‐19 infection. There are beneficial and harmful effects of *TLR* in COVID‐19 infection.	Several *TLR2* polymorphisms were found in tuberculosis patients. *TLR2* polymorphisms are a risk factor for tuberculosis infection. Yim et al. observed that *TLR‐2* deficiency makes patients more TB‐susceptible. We suggest that genetic variations that result in TLR2 deficiency can be a risk factor for TB.		[92, 93, 94, 95, 96, 97, 98, 99, 100, 101]
*TLR4*	Associated with activating gene expression and viral.	Causing HIV‐1 expression during opportunistic co‐infections.	*TLR4* mediates the (lipopolysaccharides) LPS of gram‐negative bacteria recognition.^[^ ^92^ ^]^ *TLR4* was expressed in correlation with the absolute neutrophil count.	*TLR4 is* theoretically important in COVID‐19 infection. There are beneficial and harmful effects of *TLR* in COVID‐19 infection.	*TLR4* is associated with TB pathogenesis, this stems from the number of SNPs in *TLR4* and *TLR8* in TB infected individuals compared to healthy individuals. *TLR4* and *TLR8* heterodimer formation regulates MTB immune responses.		[92, 93, 98–103]
*TLR7*		Causing HIV‐1 expression during opportunistic co‐infections.	*TLR7* and *TLR8* recognize plasmodium‐derived RNA. *TLR8* has been associated with severe infected malaria in Mali children.	*TLR7* is expressed on dendritic cells and monocytes. *TLR7/8* mediates pro‐inflammatory cytokine production.^[^ ^93^ ^]^ *TLR7* has been associated with SARS‐CoV‐2 pathogenesis. *TLR7/8* identify COVID‐19. *TLR8* is present in the lungs, and *TLR7* and *TLR8* may lead to SARS‐CoV‐1 cytokine storms.	*TLR7* has been suggested to provide MTB host cell immunity.		[93, 104–107]
*TLR8*		Causing HIV‐1 expression during opportunistic co‐infections.	*TLR8* recognizes plasmodium‐derived RNA. *TLR8* has been associated with severe infected malaria in Mali children.	*TLR8* mediates pro‐inflammatory cytokine production. *TLR8* identify COVID‐19. *TLR8* is present in the lungs, and *TLR8* may lead to SARS‐CoV‐1 cytokine storms.	*TLR4* and *TLR8* are associated with TB pathogenesis, this stems from the number of SNPs in *TLR4* and *TLR8* in TB infected individuals compared to healthy individuals. *TLR4* and *TLR8* heterodimer formation regulates MTB immune responses.		[93, 103, 106, 107, 108, 109, 110]
*TLR9*	Associated with activating gene expression and viral.	Causing HIV‐1 expression during opportunistic co‐infections.	*TLR9* facilitates recognition of the CpG motif in bacterial DNA.	*TLR9 is* theoretically important in COVID‐19 infection. There are beneficial and harmful effects of *TLR* in COVID‐19 infection.	*TLR9* is a pattern recognition receptor that facilitates MTB recognition and controls MTB‐specific T‐cell responses.	(rs5743836) A>G malaria progression and susceptibility.	[93, 111, 112, 113, 114, 115]
						(rs352140) C>T high levels in highly active antiretroviral therapy (HAART)‐naïve rapid progressors.	
Cytokines							
*IFN*	Regulates cytokines and chemokines. It is involved in innate immunity.	Aberrant *IFNG* regulation.	*IFN‐γ* was higher among individuals who were parasitemic than aparasitemic. Reduced *IFN‐α* levels were associated with severe malaria in Kenyan and Gabonese children. *IFN‐α* regulates the immune response during human malarial infections. *IFN* has differing effects on infectious diseases.	Severe COVID‐19 is associated with sustained *IFN* response. Severe COVID‐19 patients had disrupted *IFN‐α* and *β* production and downregulation of *IFN‐simulated* genes. Mossel et al. did an in vitro study and showed that *IFN‐α* and *IFN‐γ* have a synergistic effect that inhibits SARS‐CoV replication.	*IFN‐α* expression levels are higher among TB patients than uninfected individuals. In‐vitro *IFN‐α* does not restrict MTB replication intracellularly*. IFN‐γ* is associated with anti‐mycobacterial activity. *IFN‐*α compromised the activity of *IFN‐γ*. *IFN‐α* inhibited *IL‐1β* production and induced the production of *IL‐10*, which further reduced *IL‐1β*. *IFN‐α* enhances *IFN‐γ* production*. IFN‐α* and *IFN‐γ* might be associated with MTB immune escape and disease progression.		[116, 117, 118, 119, 120, 121, 122, 123, 124, 125]
*TNF*	Is associated with cell proliferation, death, survival, and differentiation.	It is important for HIV pathogenesis. In addition, HIV uses *TNF‐α* signaling pathways to expand its reservoir. HIV proteins act like and mediate the *TNF* signaling pathway.	*TNF* is known for malaria killing, however, it also is associated with severe malaria development.	When *TNF* was blocked in severe COVID‐19 patients there was reduction in lung damage and lower hospitalization. *TNF* was associated with severe COVID‐19 pathogenesis and may have a role in cytokine storms.	*TNF‐α* contributes to MTB host response. Inhibition of *TNF‐α* is associated with an elevated risk of latent TB infection reactivation. *TNF* plays an important role in the host's immune response against TB.		[126, 127, 128, 129, 130, 131]
Interleukins							
*IL1*	Inflammatory response.	The *IL‐1* family is primarily associated with innate immunity, manifested by inflammation, and functioning as a mechanism of host defense. *IL‐1* triggers innate inflammation via *IL‐1* receptors. *IL‐1a* also plays a role in acquired immunity as damage‐associated molecular patterns (DAMPs). HIV inducive *IL‐1* plays a role in HIV‐1 disease progression. *IL‐1α* and Interleukin‐1 beta (*IL‐1β)* are co‐stimulatory cytokines for T helper cells that stimulate the maturation and clonal expansion of B cells.		This *IL‐1* SNP is associated with disease severity.	*IL‐1* accumulates in the acute phase response. Increased *IL‐1β, IL‐4*, and *IL‐6* levels were associated with TBM.	(rs16944) A>G was associated with severe influenza A/H1N1 and B.	[92, 132–142]
*IL4*		Anti‐inflammatory cytokine with immune‐regulating functions such as, downregulation of HIV co‐receptors; *CCR5* and upregulation of *CXCR4*, influence transcription of the cytokine, inhibits HIV‐1 replication mostly for *CXCR4*.			Increased *IL‐4* levels were associated with TBM		[127, 138, 142–146]
*IL6*	Accumulates in acute response.	*IL‐6* starts a signaling cascade associated with the Janus kinase/signal transducer and activator of transcription (JAK‐STAT) activation pathway, promoting the transcription of genes associated with cellular signaling processes. *IL‐6* and the soluble form of the *IL‐6* receptor *(sIL‐6Rα*) determine the change from acute to chronic inflammation by changing the nature of leucocyte infiltrate. *IL‐6* stimulates the production of *IL‐1* receptor antagonists, an anti‐inflammatory mediator.	The concentration of *IL‐6* has been associated with severe malaria and death.	Higher levels of inflammatory cytokines such as *IL‐6* in bronchoalveolar lavage fluids (BALFs) have been associated with severe COVID‐19 infection compared to moderate infection.	The effect of CD4+ T cell response makes *IL‐6* crucial in the protection against murine *M. tuberculosis* infection. *IL‐6* deficiency resulted in an altered Th1 response and raised bacterial loads. *M. tuberculosis*‐infected macrophages secreted *IL‐6* which overturned the responses of uninfected macrophages to interferon *(IFN*). Elevated *IL‐6* in the lungs and increased concentrations of *IL‐1β* correlated with TB progression. *IL‐6* was shown to positively and negatively contribute to host control against *TB* infection.	Rs1800795 G>C	[41, 92, 132, 133, 137, 138, 140–142, 147–163]
*Il‐8*	*IL‐8* has specificity to neutrophils, it attracts and activates them in inflammatory regions.	The increase of *IL‐8* in the peripheral blood and lymphoid tissue of HIV‐infected individuals indicates that *IL‐8* is important in HIV‐1 pathogenesis. During early infection, *IL‐8* decreased HIV‐1 reverse transcription and viral integration.	The *IL‐8* receptor binds to *P. vivax* and is involved in inflammation.	Higher levels of inflammatory cytokines such as *IL‐8* in BALFs have been associated with severe COVID‐19 infection.	*IL‐8* is associated with TB.	rs4073 A>T increases TB susceptibility.	[164, 165, 166]
*IL‐10*	Pro‐inflammatory cytokine.	Inhibits HIV‐1 replication.		*IL‐10* is important in the COVID‐19 host response.	*IL‐10* is important in the TB host response, *it* increases the intracellular survival of mycobacterial bacilli by inhibiting phagosomal maturation, reducing nitric oxide production, and blocking IFN‐γ signaling in macrophages. Increased levels of *IL‐10* are associated with MTB susceptibility.		[143, 167–169]
*IL‐18*	*IL‐18* is associated with the activation of innate immune cells, and growing evidence in polarization of the adaptive immune system response.	*IL‐18* induces IFN production in T cells and enhances NK cytotoxic activity.	*IL‐18* is a potent proinflammatory cytokine that induces *IFN‐γ* production from Th1 cells, NK cells, and activated macrophages. *IL‐18* induces production in various cells. *IL‐18* induces severe malaria through an elevating *IFN‐γ* pathway.	In addition, *IL‐18* correlates with severe COVID‐19 and thus plays a role in inflammasome activation and pyroptosis.	A study showed an association between *IL‐18* and TB among children. *IL‐18* was said to be involved in immune response and MTB control.		[170, 171, 172, 173]
Other							
*VDR*	Transcription factor that binds to active vitamin D.	VDR mutation alters vitamin D's ability to alter immune regulation.	*VDR* has been associated with the severity of parasitemia and gametocytemia clearance in *Plasmodium vivax* malaria infections. *VDR* plays a role in the malaria immune response.	Lack of vitamin D and inactivation of *VDR* have been associated with increased respiratory syndrome in COVID‐19 individuals via a wounding response in stellate cells of the respiratory system.	Host gene polymorphisms are associated with TB resistance and susceptibility. Studies have implicated a link between vitamin D deficiency and TB susceptibility. An increase in vitamin D has shown benefits to cutaneous TB.	Rs11568820	[4, 14, 18–22, 24, 174–181]
						Rs4516035	
*MBL*	Involved in the innate immune system. Binds sugars and inters with pathogens.	Blocks the interaction between HIV and *DC‐SIGN*. In the innate immune system, *MBL* attacks glycosylated gp120 of the virus.	*MBL‐2* polymorphisms were associated with malaria disease severity.	*MBL* binds to the COVID‐19 spike protein in a glycan‐dependent manner and inhibits SARS‐CoV‐2. Thereafter, *MBL* activates the lectin pathway of complement activation. *MBL* was predicted to recognize the Omicron variant of concern (VOC). of *MBL*.	Increases phagocytosis activation of the cascade by binding to TB.	Rs1800450, C>T severe malaria susceptibility.	[23, 182–187]
*ICAM‐1*	Involved in inflammatory processes.	Enhances HIV‐1 infectivity, and makes virions resistant to neutralization with gp120‐specific antibodies.	The *ICAM‐1* Kilifi genetic variation has a possible role in severe malaria and CM pathogenesis. *ICAM‐1* Kilifi was associated with increased susceptibility to CM. *ICAM‐1* plays a role in host cell invasion as receptors or crucial accessory molecules in *Plasmodium falciparum*.	High levels of *ICAM‐1* are associated with severe COVID‐19 and thus a predictor of severe COVID‐19.	*ICAM‐1* plays a role in host cell invasion as receptors or crucial accessory molecules in *Mycobacterium tuberculosis*. *ICAM‐1* was increased in TB‐infected individuals compared to uninfected individuals. Increased *ICAM‐1* provides increased cell adhesion in TB‐infected monolayers.	Rs5498 A>G associated with severe malaria and TB susceptibility.	[188, 189, 190, 191, 192, 193]

### 2.1 VDR

The *VDR* gene is a transcription factor that binds to active vitamin D, 1,25(OH)_2_D_3_, and controls the expression of nearly 900 genes that are involved in multiple physiological functions.^[^ ^18^ ^]^ Vitamin D is involved in calcium homeostasis, cell proliferation, immunity, and cell differentiation to target tissues.^[^ ^19^ ^]^ Most vitamin D activities are applied through the nuclear *VDR*‐mediated control of target genes.^[^ ^174^ ^]^ There were 60 SNPs found in the *VDR* gene that have been associated with altered *VDR* function.^[^ ^4^, ^24^ ^]^ SNPs in the *VDR* gene have been associated with worse HIV susceptibility and disease progression. These polymorphisms may change the gene function and the role of 1,25 dihydroxy vitamin D. Some polymorphisms result in a shortened form of *VDR* protein or an abnormal receptor that interferes with signaling.^[^ ^175^, ^176^, ^177^ ^]^ *VDR* polymorphism is in strong linkage disequilibrium with other *VDR* polymorphisms. 3′ UTR polymorphisms are associated with HIV disease.^[^ ^21^ ^]^ It can be suggested that these polymorphisms might also play a role in HIV disease. Polymorphism Cdx (rs11568820) C > T is in the 5′ UTR promoter region and is involved in transcriptional activity. Polymorphism rs11568820 C > T showed an association with HIV‐1 protection.^[^ ^24^ ^]^ In Africans, the T allele frequency is 0.80, suggesting that there might be HIV protection among Africans. De la torre et al. used multilocus logistic regression analysis to show haplotypes for rs11568820 and rs4516035 polymorphisms associated with protection against HIV‐1 infection in 460 males who were HIV‐1 exposed injection drug users, of which 335 were infected and 125 uninfected (*p* = 0.0025).^[^ ^24^ ^]^ This is an indication of protective polymorphisms that individuals possess, despite their continual HIV exposure they remain HIV uninfected.

Host gene polymorphisms are associated with TB resistance and susceptibility. Studies have implicated a link between vitamin D deficiency and TB susceptibility. An increase in vitamin D has shown benefits to cutaneous TB.^[^ ^178^, ^179^ ^]^ Therefore, polymorphisms that reduce vitamin D levels are considered a risk factor for individuals with cutaneous TB. These genetic variations alter the innate and adaptive immune response and affect the *VDR* gene expression and ****VDR**** activity. 3′UTR polymorphisms had an association with TB in an Asian population. However, these polymorphisms were not associated with TB in the African and South American populations.^[^ ^14^ ^]^ This depicts that the effect of these polymorphisms varies among different ethnic groups. Therefore, targeting this gene for therapeutics will vary among ethnic groups and should be done with caution. In South Africa, the *VDR* polymorphisms, *Fok1*, *Bsm1*, *Apa1*, and *Taq1* resulted in the *F‐b‐A‐T* haplotype, which was seen as a protective factor for TB.^[^ ^14^ ^]^ The *b‐A‐T* haplotype showed protection against HIV, while the *B‐A‐t* haplotype was associated with TB susceptibility in HIV‐1‐infected individuals.^[^ ^14^, ^180^ ^]^ This indicates that due to the linkage of these polymorphisms, any changes in one of these polymorphisms could alter disease outcomes.

Lack of vitamin D and inactivation of *VDR* have been associated with increased respiratory syndrome in COVID‐19 individuals via a wounding response in stellate cells of the respiratory system.^[^ ^20^ ^]^ This suggests that genetic variations that reduce vitamin D levels are associated with increased COVID‐19 risk.

*VDR* has been associated with the severity of parasitemia and gametocytemia clearance in *Plasmodium vivax* malaria infections. *VDR* plays a role in the malaria immune response.^[^ ^22^ ^]^ We suggest that SNPs that affect the immune response might increase the risk of malaria. *VDR* has both positive and negative effects on malaria infected individuals. Therefore, targeting *VDR* as potential therapeutics for malaria might be challenging.

*VDR* gene expression is associated with disease and is dependent on ethnicity and the environment. *VDR* levels and activity differ among populations. Andrzej et al. showed that ethnicity had a significant effect on *VDR* expression and protein level. Caucasians had a higher *VDR* mRNA level, while Africans had significantly higher basal and control *VDR* protein levels.^[^ ^181^ ^]^ There was an inverse relationship between gene expression and protein level. This indicated that there was differential post‐transcriptional regulation between Africans and Caucasians, and the dynamics of *VDR* mRNA translation may differ among different populations.^[^ ^181^ ^]^ This is an indication of the differences across different ethnic groups. Despite vitamin D being important for *VDR* activity, there are multiple contributing factors to *VDR*‐related disease susceptibility.^[^ ^181^ ^]^ We suggest that future studies should include such factors, along with different ethnic groups, particularly those of African descent where the disease burden is high. This gene might be targeted differently in different ethnic groups for a particular disease. More studies are required to identify *VDR* polymorphisms that are associated with malaria. This will help us better understand the relationship between host genetics and disease.

### 2.2 MBL

Mannose‐binding lectin (*MBL*) is known as a pattern recognition molecule that participates in the innate immune system. *MBL* binds to a range of sugars, which allows interaction with various pathogens.^[^ ^182^ ^]^ *MBL* binds to HIV via glycosylated surface residues. It interacts with HIV by direct inactivation and opsonization, neutralization by complement activation, cytokine modulation, or inhibition of uptake by target cells. *MBL‐2* promoter and *MBL‐2* exon 1 genetic variations resulted in decreased protein levels and increased HIV susceptibility.^[^ ^183^ ^]^ Polymorphisms in the promoter region are likely to affect gene transcription and disrupt the normal functioning of the gene, resulting in increased disease susceptibility. In addition, polymorphisms in the exon region that code for the protein would have the same effect. *MBL‐2* structural variants are located at the first exon at codons 52, 54, and 57. *MBL‐2* 57AC (rs1800451) was associated with the risk of HIV‐associated neurocognitive disorder (HAND) severity (*p*  =  0.0009). *MBL‐2* 57AC genotype and 57C alleles were linked with susceptibility to HAND (*p*  =  0.003), and HIV disease severity (*p*  =  0.001). Haplotype ACA which consists of *MBL‐2* 52A, 54A, and 57C was significantly associated with HAND susceptibility and severity, and HIV‐1 acquisition (*p*  =  0.005).^[^ ^23^ ^]^ The T allele frequency for rs1800451 among Africans is 0.22, while in Europeans it is 0.02.^[^ ^23^, ^184^ ^]^ This genetic variation rarely occurs among Europeans, indicating that HIV‐1 susceptibility and the risk of HAND are less likely among Europeans compared to Africans. This indicates that these structural mutations in the exon region play a very important role in HIV susceptibility and severity. We suggest that more research on the exon region is required to determine the importance of these gene polymorphisms in HIV disease.

*MBL* is a calcium‐dependent serum lectin, that works with the immune system to enhance phagocytosis activation of the complement cascade, by effectively binding to mycobacteria. *MBL* deficiency results in amplified susceptibility to various infections caused by microorganisms and viruses. This indicates that genetic variations that reduce *MBL* will increase susceptibility to TB. However, another study showed that decreased *MBL* serum has been demonstrated in a *MBL* gene polymorphism at position 57 (rs1800541), which results in TB resistance.^[^ ^185^ ^]^ More research is necessary to establish the function and the impact of *MBL* polymorphisms on TB.

Furthermore, *MBL* binds to the SARS‐CoV‐2 spike protein in a glycan‐dependent manner and inhibits SARS‐CoV‐2. Thereafter, *MBL* activates the lectin pathway of complement activation. *MBL* was predicted to recognize the Omicron variant of concern (VOC). This shows the importance of *MBL* in SARS‐CoV‐2 infection. Due to the importance of *MBL*, we suggest that further studies on *MBL* and *MBL* polymorphisms are required to determine which polymorphisms possess COVID‐19 risk or protection.

*MBL‐2* polymorphisms were associated with malaria disease severity.^[^ ^186^ ^]^ Luty et al. showed a relationship between polymorphisms at codon 54 rs1800450 C > T, Glycine > Aspartic acid and codon 57 (rs1800451), C > T, Glycine > Glutamic acid, and severe malaria susceptibility.^[^ ^187^ ^]^ We suggest that because the T allele frequency is almost absent among Europeans, malaria susceptibility and severity are lower in Europeans than in Africans.

### 2.3 ICAM‐1

Intercellular adhesion molecule‐1 *(ICAM‐1)* is part of the immunoglobulin superfamily and is expressed on the vascular endothelium cell surface. *ICAM‐1* is involved in the inflammatory processes and T‐cell‐mediated host defense. It is a costimulatory molecule located on antigen‐presenting cells. They activate major histocompatibility complex (*MHC*) class II restricted T‐cells that are associated with *MHC* class I to activate cytotoxic T‐cells.^[^ ^188^ ^]^ *ICAM‐1* expression levels are directly proportional to HIV disease progression. The *ICAM‐1* cell surface influences HIV‐mediated syncytia formation and virus spread. *ICAM‐1* causes HIV to be more infectious and resistant to neutralization. We suggest that polymorphisms that reduce *ICAM‐1* expression levels might have a protective effect against HIV. *ICAM‐1* is a potential therapeutic target in HIV‐infected individuals.^[^ ^189^ ^]^ More research is required to evaluate *ICAM‐1* as a potential therapeutic target for HIV and other diseases.

*ICAM‐1* plays a role in host cell invasion as receptors or crucial accessory molecules in *Mycobacterium tuberculosis* and *Plasmodium falciparum*, respectively.^[^ ^190^ ^]^ *ICAM‐1* was increased in TB‐infected individuals compared to uninfected individuals. Increased *ICAM‐1* provides increased cell adhesion in TB‐infected monolayers.^[^ ^191^ ^]^ This indicates that polymorphisms associated with increased *ICAM‐1* increase TB susceptibility and severity. A similar effect is seen in HIV, therefore, we suggest this could be a potential therapeutic target for TB, as it is for HIV.

Similarly, high levels of *ICAM‐1* are associated with severe COVID‐19 and thus a predictor of severe COVID‐19.^[^ ^192^ ^]^ Increased *ICAM‐1* is associated with all these infectious diseases. The Kilifi (rs5491) polymorphism is an A to T allele change at position 179, this results in a methionine to lysine amino acid change which may affect the function of the protein,^[^ ^193^ ^]^ and result in an altered response to disease.

The *ICAM‐1* Kilifi polymorphism has been found in Kenyan children with severe malaria. This polymorphism has been associated with the risk of severe malaria in East Africa. However, this was not replicated in other populations in West and Central Africa such as Gambia and Malawi.^[^ ^194^ ^]^ This genetic variation has a possible role in severe malaria and CM pathogenesis.^[^ ^141^, ^142^ ^]^ Another study showed that *ICAM‐1* Kilifi was associated with increased susceptibility to CM. This polymorphism is almost absent in the European population, while found at a 20% frequency in Africans. This might contribute to the highest malaria rate among Africans. This could be due to the genetic variation selection of population genetics.^[^ ^193^ ^]^ The Kilifi polymorphism may play a similar role in TB, HIV, malaria, and COVID‐19. This polymorphism should be investigated in greater detail. Furthermore, a different *ICAM‐1* SNP on exon6 (rs5498) A > G was associated with severe malaria susceptibility in Nigerians.^[^ ^193^ ^]^ Targeting this gene in therapeutic methods will be beneficial for all the diseases mentioned.

## 3 Chemokine Receptor

### 3.1 CXCR6

Like CD8^+^ T cells, C‐X‐C Motif chemokine receptor 6 *(CXCR6)* is involved in the maintenance of natural killer T cells (NKT) and the natural killer (NK) cell population.^[^ ^195^, ^196^ ^]^ Although, *CXCR6* does not alter the cell function.^[^ ^197^ ^]^ *CXCR6* is a secondary co‐receptor for HIV that facilitates the fusion of HIV‐1 dual‐tropic strains and M‐tropic to CD4+ T cells.^[^ ^78^ ^]^ The protective *CXCR6* SNP (rs2234355) G > A has an added effect in viremic controllers. *CXCR6* expression levels were higher in viremic controls compared to healthy controllers and progressors (*p*_bonf_ < 0.0001). Although, this *CXCR6* SNP had an association with HIV‐1 disease control, it does not associate with elite controllers in black South Africans.^[^ ^198^ ^]^ This shows the different effects polymorphisms can have on different populations and at different points of disease progression. We suggest more studies with different ethnic groups are required to establish the role of this SNP and determine other SNPs that are associated with HIV. Precision medicine should be investigated when targeting this gene which is variable among different ethnic groups.

*CXCR6* influences the inflammatory response mechanism. *CXCR6* deficiency results in increased *M. tuberculosis* control. In the lung, *CXCR6* is upregulated more on human bronchoalveolar lavage (BAL)‐derived and lung T‐lymphocytes than in the peripheral blood. Increased *CXCR6* expression on lung CD8^+^ T‐lymphocytes correlated with chronic obstructive pulmonary severity.^[^ ^199^, ^200^, ^201^ ^]^ This indicates that polymorphisms that affect *CXCR6* function and decrease its expression will result in less severe TB and better TB control. Research on which polymorphisms lead to differential *CXCR6* expression levels will be beneficial for therapeutic strategies against TB.

*CXCR6* regulates parts of lung memory CD8+ T cells during an unrelenting immune response to aero‐pathogens, including other coronaviruses.^[^ ^202^ ^]^ *CXCR6* is a tissue‐residence gene that is found in higher quantities in expanded CD8+ T cells compared to non‐expanded cells in moderate SARS‐CoV‐2 infections.^[^ ^203^ ^]^ High amounts of total CD8+ T cells are found in moderate SARS‐CoV‐2 infection compared to severe infection. C‐X‐C motif ligand 16 (*CXCL16*), which produces a byproduct that binds *CXCR6*, was expressed more in moderate infection than in severe SARS‐CoV‐2 infection. The *CXCL16* chemokine and *CXCR6* receptors are associated with the migration and survival of NKT and innate lymphoid cells (ILC). They are elevated in hospitalized COVID‐19 patients.^[^ ^204^ ^]^ *CXCR6* needs to be studied in greater detail to determine its role in COVID‐19 infected individuals.

In addition, *CXCR6* is important in maintaining protective resident memory T cells in the liver following malaria infection in mice. *CXCR6* deficient CD8+ T cells decrease in the liver and prevent malaria inhibition.^[^ ^205^, ^206^ ^]^ Therefore, functional mutations in *CXCR6* will likely result in the risk of malaria severity over time. The frequency of SNPs and haplotypes differ significantly in different ethnic groups. This was observed in a South African population among Africans and Caucasians. Genetic studies with human subjects from Africa, with the greatest genetic diversity, are most beneficial to determine therapeutic targets for all infectious diseases.

### 3.2 CX3CR1

CX3C chemokine receptor 1 *(CX3CR1)* is likely to be co‐receptors for HIV‐1 according to its functionality in vitro. *CX3CR1* is associated with cell adhesion and migration. Mutations that occur in some of these genes have shown an association with HIV disease progression. High levels of *CX3CR1* expression are associated with HIV infection (*p* = 0.002).^[^ ^25^ ^]^

Like HIV, the *CX3CR1* expression levels on monocytes are elevated among latent TB infections.^[^ ^56^ ^]^ *CX3CR1* is vital for the development of atherosclerotic plaque because it assists with the recruiting of non‐classical monocytes.^[^ ^55^ ^]^ Future studies with large cohorts and varying ethnicities on *CX3CR1* are needed to determine its function in TB. Due to the similar effect observed in TB and HIV, it could be beneficial for the high rate of co‐infection with TB and HIV in Africa and other countries.

*CX3CR1* expression was impaired among individuals who had contrary artery disease and SARS‐CoV2. *CX3CR1* mediates non classical monocyte migration along with endothelial cells in the vascular system for antiviral immune response. *CX3CR1* is a candidate gene for COVID‐19 severity and SARS‐CoV‐2 susceptibility. The role of polymorphisms in this gene is unknown.^[^ ^57^ ^]^ The gap in the research on the effect of these polymorphisms needs to be addressed as COVID‐19 still exists globally. COVID‐19 relationship with various other diseases and comorbidities makes it a challenging study.

High *CX3CR1* and *CCR2* on CD14 monocytes limit parasitic growth through antibody dependent cellular inhibition activity.^[^ ^58^ ^]^ Similarly, in another study, high *CX3CR1* expressing cells were associated with better survival.^[^ ^59^ ^]^ *CX3CR1* has differing effects on different infectious diseases. More studies are needed to unravel the roles of polymorphisms in *CX3CR1*.

### 3.3 CCR2

C‐C motif chemokine receptor 2 (*CCR2*) is a G protein coupled receptor. The *CCR2* and *CCL2* interaction is important for inflammatory diseases and inflammation. It is also associated with innate immune response by recruiting monocytes into the inflammation site. *CCR2* alters the location of *CCR5* and *CXCR4* on the cell surface and regulates heterodimerization. Furthermore, the monoclonal antibody *CCR2‐01* disrupts HIV replication by heterooligomerization induction of *CCR2* with *CXCR4* and *CCR5* co‐receptors.^[^ ^27^ ^]^ They also form dimers with *CCR5* and *CXCR4*.^[^ ^28^, ^29^, ^30^ ^]^

Studies show that *CCR2* contributes to the defense against *M. tuberculosis* (MTB). This was shown in a study with *CCR2*‐deficient mice.^[^ ^32^ ^]^ Studies with human participants are required to determine the effect of *CCR2* on TB. These studies should also include individuals of varying ethnicities to ensure that target genes are ethnic‐specific to ensure an effective response.

*CCR2* is associated with the monocyte recruitment and infiltration of these cells in the lungs of COVID‐19 infected individuals. *CCR2* may be protective in SARS‐CoV‐infected dendritic cells. *CCR2* reduced viral load in SARS‐CoV‐2 infected mice.^[^ ^31^ ^]^ This suggests that mutations that reduce the *CCR2* expression and function might increase the viral load. More studies with human subjects are needed to unravel the function of *CCR2* in COVID‐19.

*CCR2* deficient mice have persistent parasitemia in malaria.^[^ ^33^ ^]^ *CCR2* deficient mice inhibit inflammatory monocyte recruitment to the site of infection.^[^ ^34^ ^]^ *CCR2* has a protective effect on all four infectious diseases, therefore *CCR2* is an important gene to study in different populations, particularly in the African population. More mutations that influence *CCR2* need to be identified.

### 3.4 CCR5

C‐C motif chemokine receptor 5 (*CCR5*) is located on *3p21.31. CCR5* is upregulated by proinflammatory cytokines and is mainly expressed in memory T‐cells, macrophages, and dendritic cells. *CCR5* is the main coreceptor used by HIV‐1 and HIV‐2 responsible for viral transmission. *CCR5* plays a vital role in HIV pathogenesis.^[^ ^35^ ^]^

Additionally, *CCR5* plays an essential role in cell activation and migration in immune responses against TB. A study that consisted of 450 TB patients and 306 healthy controls showed that *CCR5* promoter polymorphisms were found to be associated with pulmonary TB and TB progression in the Chinese Han population.^[^ ^36^ ^]^ *CCR5* is highly expressed on T helper (Th) 1 cells. Th1 responses play a critical role in TB immunity.^[^ ^37^ ^]^ There are elevated *CCR5* levels in Th cells in pulmonary TB patients.^[^ ^37^, ^38^ ^]^ *CCR5* expression is increased in individuals with active TB.^[^ ^39^, ^40^ ^]^ In addition, *CCR5* ligands are also increased during TB^[^ ^38^, ^39^, ^40^, ^41^ ^]^ and cell‐mediated immunity (CMI) is a central determinant of TB resistance. The ligand of *CCR5* ^[^ ^42^ ^]^ influences CMI.^[^ ^43^, ^44^ ^]^ *CCR5*‐HHD haplotype is found more in African ancestry. Further studies on its influence on HIV infection and TB susceptibility contributing to the growing burden of HIV and TB in Africa are necessary.^[^ ^43^ ^]^

*CCR5* is expressed in macrophages and T cells acting as a co‐receptor for macrophage‐tropic viruses and plays an important role in SARS‐CoV‐2 infection.^[^ ^45^ ^]^ The rs9845542 and rs12639314 variants of *CCR5* were associated with severe COVID‐19 disease.^[^ ^26^ ^]^ *CCR5* plays a role in COVID‐19 severity.^[^ ^46^ ^]^ The *CCR5Δ32* deletion leads to reduced expression, playing a protective role against SARS‐CoV‐2 infection.^[^ ^47^, ^48^ ^]^ This effect has also been observed in HIV.^[^ ^46^ ^]^ This suggests that reduced expression of *CCR5* is beneficial against TB, HIV, and COVID‐19.

*CCR5* was associated with adverse effects caused during pregnancy with malaria infection. *CCR5* deficiency increases maternal parasitemia. More information on the role of *CCR5* in malaria needs to be better understood.^[^ ^49^ ^]^ *CCR5* is a very important gene that plays an essential role in all the above diseases. Studies that focus on *CCR5* are required in an African population. There are various successful stories regarding targeting *CCR5* in HIV infected individuals. While this is promising for future therapeutics targeting this gene is also associated with various other diseases, which makes it a good target for individuals who are infected with one or more of the other infectious diseases.

### 3.5 CXCR4

C‐X‐C motif chemokine receptor 4 (*CXCR4*) regulates and expresses T cell migration along gradients of *CXCL12*. *CXCR4* is a chemokine coreceptor that allows X4 HIV viral strain entry but reduces R5 viral entry. *CXCR4* expression is higher among TB/HIV co‐infected individuals than HIV infected individuals Increased *CXCR4* expression is associated with TB in alveolar macrophages.^[^ ^51^ ^]^

High levels of *CXCR4* direct cells to destroy lung tissue.^[^ ^50^ ^]^ In a mixed cohort of Latins, Asians, and whites, fatal COVID‐19 is associated with elevated *CXCR4* in bystander T cells.^[^ ^52^ ^]^ Interestingly, another study with Caucasian participants showed that low levels of *CXCR4* were associated with COVID‐19.^[^ ^53^ ^]^ Due to contradictory results, more studies are required to determine the function of *CXCR4*. This might also be variation among different ethnic groups.

*CXCR4* increases calcium levels which influence sporozoite transformation into exoerythrocytic forms which is necessary for parasite progression in the liver. Eliminating *CXCR4* inhibits the development of *P. falciparum* in the liver.^[^ ^54^ ^]^ Increased *CXCR4* is detrimental to protection against these infectious diseases. Further studies should be performed among individuals of different ethnicities. Very few studies have evaluated the differences between cytokines across different ethnic groups. We are unable to highlight these differences with certainty.

## 4 Chemokine

### 4.1 SDF‐1

Stromal cell‐derived factor 1 (*SDF‐1*) is a chemokine protein that has a wide range of functions, one being the ability to control the trafficking of leukocytes.^[^ ^60^ ^]^ *CXCL12/SDF‐1* prevented the accumulation of newly reverse‐transcribed HIV proviral DNA, required for productive infection. *SDF‐1* did not inhibit viral replication.^[^ ^61^ ^]^ Furthermore, *SDF‐1* is a diagnostic marker for TB and correlates with severe TB.^[^ ^60^ ^]^

*SDF‐1* is upregulated early post‐SARS‐CoV‐2 infection. The *SDF‐1* chemokine and the *CXCR4* receptor are responsible for bone marrow homing, it is upregulated and remains steady post‐SARS‐CoV‐2 infection. More studies with a large cohort of varying ethnicities are necessary to determine the effects of *SDF‐1* on COVID‐19 disease.

Furthermore, high *SDF‐1* levels in African children were associated with acute malaria compared to healthy children.^[^ ^62^ ^]^ There are different effects of *SDF‐1* among these diseases. Therefore, targeting this gene should be disease‐specific and future and current disease exposures should be considered. There is a gap in the role of *SDF‐1* and its polymorphisms in infectious diseases.

### 4.2 CCL5

Regulated on activation, normal T expressed and secreted (*RANTES*)/chemokine C‐C motif ligand (*CCL5)* is a *CCR5* ligand that is involved in the migration of macrophages and NK cells as well as the T cell/ dendritic cells (DCs) interaction. *CCL5* inhibits *CCR5* HIV infections. Genetic variations in the *CCL5* promoter region are associated with more *CCL5* transcription, resulting in delayed HIV disease progression.^[^ ^84^ ^]^ Gonzalez et al. indicated a large amount of variation in the genotype frequencies between races, and different disease effects depending on ethnicity. The diverse spread of *RANTES* haplotypes (AC, GC, and AG) was associated with population‐specific HIV‐1 transmission and varied disease outcomes. Individuals with a homozygosity AC haplotype were associated with an increased risk of acquiring HIV‐1 and disease progression in European Americans, but not in African Americans. Although, there was a higher prevalence of the AC haplotype in Africans compared to non‐Africans.^[^ ^85^ ^]^ This indicates that the same haplotype can have differing effects on different populations. Therefore, taking ethnicity into account is of great importance. In a Japanese cohort, AG‐containing RANTES haplotype pairs were associated with delayed HIV disease progression; however, the AG haplotype is infrequent in some of these populations.^[^ ^85^ ^]^

*CCL5* plays a key role in co‐stimulation of T cell proliferation and *RANTES* activation in anti‐mycobacterial immunity. Studies have shown a significant relationship between *RANTES* polymorphisms and an increased TB risk.^[^ ^86^ ^]^ *RANTES* expression was higher in PTB patients than in healthy individuals (*p* < 0.05). In the North central Indian tribe, Sahariya with high TB prevalence showed that the *CCL5* SNP (rs2107538) C > T resulted in reduced *CCL5* expression, and was found in PTB cases and therefore significantly associated with increased PTB susceptibility.^[^ ^87^ ^]^ These studies show that an increased and decreased *CCL5* expression was associated with PTB. We suggest that the differing results could be ethnic‐specific. More research is required to unravel the role of *CCL5* and *CCL5* polymorphisms and their impact on TB.

*CCL5* is upregulated in early post‐SARS‐CoV‐2 infection. In addition, high levels of *CCL5* is found in critically ill COVID‐19 patients compared to healthy and moderately ill COVID‐19 patients.^[^ ^88^ ^]^ However, Perez‐Garcia et al. showed that high levels of SARS‐CoV‐2 viral load and low levels of *CCL5* were associated with ICU patients.^[^ ^89^ ^]^ This suggests that more information is needed to edify the role of *CCL5* in COVID‐19 disease. We suggest that the inconsistent *CCL5* level could be ethnic‐specific or patient‐specific. In addition, co‐morbidities and various other factors needed to be accounted for when comparing these studies.

Malaria outcomes are influenced by the role of *RANTES* in host immunity. Low levels of *RANTES* were associated with CM in Ugandan children.^[^ ^90^ ^]^ Bujarbaruah et al. observed similar results, showing low levels of *RANTES* were associated with severe malaria and high *RANTES* was associated with malaria recovery and uncomplicated malaria.^[^ ^91^ ^]^ *RANTES* variations affect protein production and alter host immunity.^[^ ^91^ ^]^ We suggest that polymorphisms resulting in high expression levels of *RANTES* result in better immunity and could be beneficial to individuals with malaria. Further studies are required to identify *RANTES* polymorphisms that are associated with malaria.

### 4.3 CCL3

In the presence of a pathogen, macrophage inflammatory protein one alpha (*MIP‐1a*)/ chemokine (C‐C motif) ligand 3 (*CCL3*) is involved in the differentiation and migration of effector T cells resulting in inflammation.^[^ ^71^ ^]^ *CCL3* is involved in the recruitment of neutrophils and monocytes, and the activation of inflammatory signaling.^[^ ^72^ ^]^ *CCL3* is a beta‐chemokine that possesses anti‐HIV‐1 characteristics and thus is inversely associated with HIV disease progression.^[^ ^73^ ^]^ *CCL3* is produced by CD8+ T cells. The *CCL3* gene duplication is known as chemokine (C‐C motif) ligand three like 1 (*CCL3L1*).^[^ ^74^, ^75^ ^]^ Gene duplication is referred to as copy number variation (CNV), this is when a portion of the gene is repeated. *CCL3L1* is the most effective inhibitor of *CCR5* and HIV‐1 infection caused by the R5 strain.^[^ ^76^ ^]^ The copy number of *CCL3L1* varies among ethnic groups and is prevalent among the African population.^[^ ^77^ ^]^ Surprisingly, this genetic variation is prevalent among Africans where HIV prevalence is the highest. This indicates that multiple factors play a role in HIV acquisition. Low levels of *CCL3L1* were demonstrated in acquired immune deficiency syndrome (AIDS) subjects, indicating that high levels of these chemokines are beneficial to the host. The copy number of *CCL3L1* influences HIV‐1 susceptibility.^[^ ^78^ ^]^ *CCL3L1*, an *MIP‐1* isoform, has the potential to block viral entry.^[^ ^79^ ^]^ Saha et al. demonstrated that high levels of MIP‐1a and MIP‐1b were produced in long‐term nonprogressors (LTNPs).^[^ ^80^ ^]^ The degree of *CCL3L1* expression can affect HIV infection by; internalizing *CCR5* receptors, subsiding *CCR5* expression levels on the cell surface, altering anti‐viral responses via leukocyte trafficking, and preventing *CCR5* and HIV‐1 gp120 binding.^[^ ^77^, ^81^ ^]^ Therefore, CNV is directly proportional to HIV disease. *CCL3* polymorphisms are associated with HIV resistance or susceptibility.^[^ ^82^ ^]^

*CCL3* is a leucocyte activating chemokine that is involved in TB restriction. High levels of *CCL3* were detected in PTB when compared to latent TB (LTB) and healthy control (HC) patients.^[^ ^83^ ^]^ As previously mentioned, *CCL3L1* is more prevalent in the African population. We suggest that this might be attributed to the high TB prevalence in Africa.

Similarly, *CCL3* chemokines were expressed at higher levels in the lung macrophages of severe COVID‐19 patients.^[^ ^72^ ^]^ This suggests that polymorphisms are associated with increased *CCL3* levels. Therefore, increased *CCL3* results in detrimental COVID‐19 effects.

The *CCL3* levels were increased in CM‐infected mice.^[^ ^70^ ^]^ In addition, elevated levels of monocyte‐attracting *β*‐chemokines *CCL3* have been found in malaria‐infected placentae.^[^ ^64^ ^]^ More research in humans is required to determine the function of *CCL3* and CNV in TB, malaria, and COVID‐19. High levels of *CCL3* are harmful to malaria, COVID‐19, and TB‐infected individuals but beneficial for HIV‐infected individuals.

Previous studies of *CCL3L1* CNV and its association with disease susceptibility have been found in various populations such as European, Japanese, African, and Korean. Copy number distribution differs among ethnic groups. Jamaluddin et al. suggested that ethnic groups influence genetic variation due to Malaysians having significantly different *CCL3L1* copy numbers among different Malaysian populations. This was also seen in the African population.^[^ ^16^ ^]^

### 4.4 CCL2

The chemokine (C‐C motif) ligand 2 (*CCL2)* attaches to *CCR2*. *CCL2* is a pro‐inflammatory chemokine, and *CCR2* plays a role in monocyte migration during inflammation. *CCL2* drives the T helper 2 (Th2) immune response.^[^ ^65^ ^]^ This chemokine regulates immune cell movement to the site of HIV infection and cell activation; therefore, they are associated with HIV disease progression.^[^ ^66^ ^]^ Within the *CCL2* gene, the H7 haplotype (*CCL2*‐*CCL7*‐*CCL11*) has been found to reduce HIV susceptibility.^[^ ^67^ ^]^ The H7 haplotype was significantly elevated (*p*  = 0.005–0.01) in uninfected exposed European‐Americans.^[^ ^68^ ^]^ We suggest that genetic variations in haplotype 7 influence the risk of HIV‐1 infection.

*CCL2* is associated with TB. Genetic variations in this gene have shown an increase in TB susceptibility. Feng et al. found an association between *CCL2* SNP (rs4586), resulting in a T > C allele change, and pediatric TB in Han Chinese males, indicating that gender may affect TB susceptibility even in children.^[^ ^69^ ^]^ The homozygous T genotype associated with reduced cerebrospinal fluid (CSF) and mononuclear leukocyte (ML) count suggests significance, and potential to assist with tuberculosis meningitis (TBM) assessment in serious cases.^[^ ^69^ ^]^ This polymorphism is common among Africans, it might contribute to the high TB rates in Africa. This polymorphism should be studied in more detail in an African population.

The *CCL2* chemokine is involved in the migration of inflammatory monocytes. It is produced by alveolar macrophages, T cells, and endothelial cells. They also recruit mast cell progenitors and influence the accumulation of neutrophils and procollagen synthesis through fibroblasts.^[^ ^31^ ^]^ In addition, *CCL2* chemokines were expressed at higher levels in lung macrophages of severe COVID‐19 patients. *CCL2* was upregulated early post‐SARS‐CoV‐2 infection. Higher *CCL2* levels were detected in symptomatic individuals.^[^ ^31^ ^]^ We suggest that polymorphisms that decrease the levels of *CCL2* may be protective against COVID‐19.

High levels of *CCL2* were observed in *P. vivax* infections.^[^ ^63^ ^]^ Similarly, elevated *CCL2* has been found in malaria‐infected placentae.^[^ ^64^ ^]^ We suggest that decreased levels of *CCL2* are beneficial to malaria‐infected individuals. *CCL2* is involved in the immune response to HIV, TB, malaria, and COVID‐19. It is important to consider and conduct research on *CCL2* gene distribution in different ethnic groups.

## 5 Cytokines

### 5.1 TNF

Tumor necrosis factor (*TNF*) is a cytokine that has various roles such as cell proliferation, death, survival, and differentiation. *TNF‐α* is important for HIV pathogenesis. In addition, HIV uses *TNF‐α* signaling pathways to expand its reservoir. HIV proteins act like and mediate the *TNF* signaling pathway.^[^ ^126^ ^]^ This poses a risk factor for HIV.

*TNF‐α* plays a vital contribution to MTB host response.^[^ ^127^ ^]^ Inhibition of *TNF‐α* is associated with an elevated risk of latent TB infection reactivation.^[^ ^128^ ^]^ This indicates that *TNF* is important for the prevention of latent TB reactivation. This seems to be an important therapeutic target for TB. Future research should analyze the effect of this gene in detail to determine the effects on inhibition and which polymorphisms result in inhibition.

When *TNF* was blocked in severe COVID‐19 patients there was reduction in lung damage and lower hospitalization.^[^ ^129^ ^]^ *TNF* was associated with severe COVID‐19 pathogenesis and may have a role in cytokine storms.^[^ ^130^ ^]^ *TNF* has a detrimental effect on COVID‐19.

*TNF* is known for malaria killing; however, it also is associated with severe malaria development.^[^ ^131^ ^]^ *TNF* has differing effects on different diseases. *TNF* is beneficial to TB and malaria. However, it poses a detrimental effect on HIV and COVID‐19. Targeting such a gene as a potential therapeutic for a particular disease such be done with caution of future and current exposure to other diseases.

### 5.2 IFN

*IFN* is made up of three groups, type I, type II, and type III. *IFN* regulates cytokines and chemokines. Type I *IFN*, *IFN*‐*α*, and *β* are important for innate immunity against viruses and inhibits HIV‐1 replication. *IFN* disrupts HIV infection and HIV disease progression.^[^ ^121^ ^]^ Type II *IFN‐γ* inhibits HIV‐1 entry into macrophages.^[^ ^116^ ^]^ In advanced stages of HIV infection when *IFN‐γ* reduces, viral replication persists.^[^ ^117^ ^]^ *IFN* has a protective effect against HIV. Preventing the reduction of *IFN* might prevent advanced stages of HIV.

*IFN‐α* expression levels are higher among TB patients than uninfected individuals. In vitro *IFN‐α* does not restrict MTB replication intracellularly*. IFN‐γ* is associated with anti‐mycobacterial activity. *IFN‐α* compromised the activity of *IFN‐γ*.^[^ ^122^ ^]^ *IFN‐α* inhibited *IL‐1β* production and induced the production of *IL‐10*, which further reduced *IL‐1β*. *IFN‐α* enhances *IFN‐γ* production*. IFN‐α* and *IFN‐γ* might be associated with MTB immune escape and disease progression.^[^ ^118^ ^]^

Severe COVID‐19 is associated with sustained *IFN* response. Severe COVID‐19 patients had disrupted *IFN‐α* and *β* production and downregulation of *IFN*‐simulated genes.^[^ ^123^ ^]^ Mossel et al. did an in vitro study and showed that *IFN‐α* and *IFN‐γ* have a synergistic effect that inhibits SARS‐CoV replication.^[^ ^119^ ^]^

*IFN‐γ* was higher among individuals who were parasitemic than aparasitemic.^[^ ^120^ ^]^ Reduced *IFN‐α* levels were associated with severe malaria in Kenyan and Gabonese children.^[^ ^124^, ^125^ ^]^ *IFN‐α* regulates the immune response during human malarial infections.^[^ ^124^ ^]^ *IFN* has differing effects on infectious diseases. We suggest that *IFN* types I and II should be studied together on different ethnic groups.

## 6 Interleukin (*IL*) 1, 4, 6, 8, 10, 18

Cytokines are proteins produced by cells of the immune system that detect target cells and interact with them. This interaction triggers a specific response that maintains immune homeostasis. In response to infections and tissue damage, *IL‐6*, a pleiotropic cytokine is produced.^[^ ^147^ ^]^ The *IL‐6* cytokine is produced by cells such as; mast cells, macrophages, dendritic cells, and T and B cells at the site of inflammation.^[^ ^148^ ^]^ Once specific receptors are targeted, *IL‐6* starts a signaling cascade associated with the Janus kinase/signal transducer and activator of transcription (JAK‐STAT) activation pathway,^[^ ^149^ ^]^ promoting the transcription of genes associated with cellular signaling processes.^[^ ^150^ ^]^ *IL‐6* and the soluble form of the *IL‐6* receptor (*sIL‐6Rα*) determine the change from acute to chronic inflammation by changing the nature of leucocyte infiltrate. *IL‐6* stimulates the production of *IL‐1* receptor antagonists, an anti‐inflammatory mediator.^[^ ^132^, ^133^ ^]^ *IL‐1* shares similar functions to toll‐like receptors (TLRs). The *IL‐1* family is primarily associated with innate immunity, manifested by inflammation, and functioning as a mechanism of host defense. *IL‐1* triggers innate inflammation via *IL‐1* receptors.^[^ ^134^ ^]^ *IL‐1a* also plays a role in acquired immunity as damage‐associated molecular patterns (DAMPs).^[^ ^135^ ^]^ HIV inducive *IL‐1* and *IL‐6* play a role in HIV‐1 disease progression. *IL‐1α* and Interleukin‐1 beta (*IL‐1β)* are co‐stimulatory cytokines for T helper cells that stimulate the maturation and clonal expansion of B cells.^[^ ^136^ ^]^ *IL‐4* is a T helper cell type 2 (Th2) cytokine with co‐stimulatory activity for T and B cells. It also upregulates *CCR5* and down‐regulates *CXCR4*, although its impact on viral entry is unknown. *IL‐4* presents stimulatory and anti‐HIV activity.^[^ ^143^ ^]^ *IL‐4*‐dependent also prevents HIV‐1 replication; however, this is cell‐dependent and mostly affects X4‐tropic strains. 589T/rs2243250 *IL‐4* SNP impacts cytokine transcription and has been associated with disease progression in certain cohorts.^[^ ^144^, ^145^ ^]^ This mutation is prevalent in Europeans. Previous studies showed that the *IL‐4* receptor, a chain gene has a significant association with disease progression and susceptibility to HIV‐1 infection. However, for HIV susceptibility it depends on the route of transmission.^[^ ^146^ ^]^ *IL‐4* presents stimulatory and anti‐HIV activity.^[^ ^143^ ^]^

*IL‐8* is a chemoattractant cytokine produced by tissue and blood cells. *IL‐8* has specificity to neutrophils, it attracts and activates them in inflammatory regions.^[^ ^164^ ^]^ The increase of *IL‐8* in the peripheral blood and lymphoid tissue of HIV‐infected individuals indicates that *IL‐8* is important in HIV‐1 pathogenesis. During early infection, *IL‐8* decreased HIV‐1 reverse transcription and viral integration.^[^ ^165^ ^]^ Polymorphisms that affect the normal functioning of *IL‐8* can be considered a risk factor for HIV.

*IL‐10* is a powerful anti‐inflammatory cytokine. *IL‐10* 5′A is associated with decreased *IL‐10* expression that limits infection and accelerates AIDS. Rs1800896, rs1800872, and rs2266590A have been associated with HIV disease,^[^ ^167^ ^]^ while rs2266590A is known to have a protective effect.^[^ ^168^ ^]^ In an Indonesia cohort, HIV‐infected individuals heterozygous for rs1518111 and rs1800872 have been associated with decreased CD4+ T cell count.^[^ ^169^ ^]^ Rs1800896 and rs1800872 occur more frequently in Africans than in Europeans. In contrast, rs2266590A is less frequent among Africans. *IL‐10* is a T helper cell type 2 (Th2) cytokine with co‐stimulatory activity for T and B cells. It also upregulates *CCR5* and down‐regulates *CXCR4*, although its impact on viral entry is unknown.^[^ ^143^ ^]^ *IL‐18* is associated with the activation of innate immune cells, and growing evidence in polarization of the adaptive immune system response. In addition, it assists with the Th1 response and the *IFNG* secretion.^[^ ^170^ ^]^ Caspase‐1 activation mediates the cleavage of the pro*‐IL‐1B* and pro‐*IL‐18* molecules, resulting in an inflammatory process after HIV infection.^[^ ^171^ ^]^ *IL‐8* genetic variants were better immunological responses to HIV.^[^ ^172^ ^]^

*IL‐6* and *IL‐1* accumulate in the acute phase response.^[^ ^137^ ^]^ Increased *IL‐1β, IL‐4*, and *IL‐6* levels were associated with TBM, however, *IL‐4* and *IL‐6* levels were not significantly different between TBM and individuals without meningitis and CNS infection.^[^ ^138^ ^]^ The effect of CD4+ T cell response makes *IL‐6* crucial in the protection against murine *M. tuberculosis* infection.^[^ ^151^ ^]^ *IL‐6* deficiency resulted in an altered Th1 response and raised bacterial loads.^[^ ^152^ ^]^ This indicates the importance of *IL‐6* in the immune response against TB. Therefore, any polymorphism that results in *IL‐6* deficiency will be a risk factor for TB‐infected individuals. In a study, *M. tuberculosis*‐infected macrophages secreted *IL‐6* which overturned the responses of uninfected macrophages to interferon (IFN).^[^ ^153^ ^]^ Elevated *IL‐6* in the lungs and increased concentrations of *IL‐1β* correlated with TB progression.^[^ ^41^ ^]^ *IL‐6* was shown to positively and negatively contribute to host control against *TB* infection.^[^ ^207^ ^]^ The reduced (interleukin 6 receptor) *IL‐6R* expression in TB was associated with decreased T helper cell 17 (Th17) response. *IL‐6/IL‐6R* polymorphism has been associated with susceptibility and severity of a wide range of diseases.^[^ ^154^ ^]^ *IL‐8* is associated with TB. The *IL‐8* (rs4073) SNP, results in an A > T allele change at position 251, which increases TB susceptibility.^[^ ^166^ ^]^ This SNP is more common among Europeans than Africans. This could be due to the natural selection of protective genes in the African population due to the high rates of TB in Africa. *IL‐10* is important in the COVID‐19 and TB host response in diverse ways.^[^ ^127^ ^]^ Bonecini‐Almeida et al. showed elevated *IL‐10* in BAL lung samples of TB patients.^[^ ^208^ ^]^ A study showed an association between *IL‐18* and TB among children. *IL‐18* participated in immune response and MTB control.^[^ ^172^ ^]^ *IL‐10* is a human cytokine synthesis inhibitory factor (CSIF), that increases the intracellular survival of mycobacterial bacilli by inhibiting phagosomal maturation, reducing nitric oxide production, and blocking *IFN‐γ* signaling in macrophages.^[^ ^209^ ^]^ Increased levels of *IL‐10* are associated with MTB susceptibility.^[^ ^127^ ^]^

Higher levels of inflammatory cytokines such as *IL‐8*, *IL‐6*, and *IL‐1β* in bronchoalveolar lavage fluids (BALFs) have been associated with severe SARS‐CoV‐2 infection compared to moderate infection. In addition, *IL‐1β* and *IL‐6* were expressed at higher levels in lung macrophages of severe COVID‐19 patients. *IL‐1* and *IL‐6* could be associated with cytokine storms and COVID‐19 complications such as venous thrombosis. Similarly, *IL‐4* and *IL‐10* are found at higher levels in severe COVID‐19 patients than in healthy individuals, especially during cytokine storms.^[^ ^127^ ^]^ In Iran, *IL‐1 β* (HGNC:5992) (rs16944) A > G was associated with severe influenza A/H1N1 and B.^[^ ^139^ ^]^ This SNP is less frequent among Africans when compared to Europeans. This might be one possible contributing factor to the lower COVID‐19 death and infection rate among Africans than Europeans. Another *IL‐6* SNP (rs1800797) A > G at the promoter region along with previously mentioned polymorphisms^[^ ^155^ ^]^ could be considered for studying COVID‐19 disease progression. *IL‐6* promoter activity reaches its highest level when the p65 and N proteins are present, these proteins have a synergetic effect on *IL‐6* activation. The nuclear factor kappa B (NF‐κB) binding site polymorphism of the *IL‐6* promoter (pIL6‐luc‐651ΔNF‐κB) eliminates the effect of the p65 and N proteins on *IL‐6* promoter activation.^[^ ^156^, ^157^ ^]^ In addition, *IL‐18* correlates with severe COVID‐19 and thus plays a role in inflammasome activation and pyroptosis.^[^ ^173^ ^]^

Griffiths et al.^[^ ^140^ ^]^ showed that a cluster of genes containing *IL‐6R* and *IL‐1R2* was expressed in correlation with the absolute neutrophil count. This gene region consisted of genes associated with mediators of innate and adaptive immunity.^[^ ^140^ ^]^ Differences in gene expression suggest that neutrophil response plays a role in acute malarial infection.^[^ ^141^ ^]^ Activation of the *TLR* pathways results in the secretion of pro‐inflammatory cytokines, such as *IL‐1* and *IL‐6*. The *IL‐8* receptor binds to *P. vivax* and is involved in inflammation.^[^ ^92^ ^]^ In malaria‐infected adults, *IL‐4* expression levels were reduced.^[^ ^142^ ^]^ The concentration of *IL‐6* has been associated with severe malaria and death. The *IL‐6* rs1800795, resulting in a G > C allele change at position 174/176 was associated with increased *IL‐6* expression in individuals with developing acute phase reactions.^[^ ^158^, ^159^, ^160^, ^161^ ^]^ This SNP is more prevalent in the African population than the European population. We suggest that this SNP might contribute to the higher malaria rate in Africa than in Europe. In Mali, the frequency of *IL‐6* CG/GG was higher than in non‐Fulanis, who had increased malaria susceptibility, in symptomatic and asymptomatic malaria.^[^ ^162^, ^163^ ^]^ *IL‐18* is a potent proinflammatory cytokine that induces *IFN‐γ* production from Th1 cells, NK cells, and activated macrophages. *IL‐18* induces *IL‐4* and *IL‐13* production in various cells. *IL‐18* induces severe malaria through an elevating *IFN‐γ* pathway.^[^ ^172^ ^]^

Hick et al. showed an appreciable difference in gene expression levels of 300 genes among Caucasians, Africans, Hispanics, and Asians in the United States. There was also an overlap in differential expression levels and patterns of gene expression among these ethnic groups.^[^ ^210^ ^]^ This indicates changes in cytokine levels across ethnic groups and highlights the specialized differences.

## 7 Receptors

### 7.1 Toll‐Like Receptors (*TLRs*) 2, 4, 7, 8, 9

*TLRs* are involved in the innate immune response against pathogens by regulating the degree of viral replication.^[^ ^211^, ^212^ ^]^ *TLRs* identify motifs presented by pathogens and trigger inflammation.^[^ ^135^ ^]^ HIV directly activates *TLRs*, while opportunistic pathogens indirectly activate *TLRs*, impacting disease progression. Signals conciliated by pathogens can activate HIV transcription via cytokines, induce cell factor expression, and activate viral long terminal repeats.^[^ ^211^ ^]^ *TLR2, TLR4*, and *TLR9* are associated with activating gene expression and viral replication during HIV infection and contribute to HIV severity through an antigenic group (Ag) and (5′‐Cytosine‐phosphate‐Guanine‐3′) CpG induction.^[^ ^98^, ^99^, ^100^, ^101^ ^]^ *TLR9* has been associated with modulating the extent of viral replication. *TLR9* SNP (rs352140) C > T is found at high levels in highly active antiretroviral therapy (HAART)‐naïve rapid progressors compared to normal progressors.^[^ ^111^ ^]^ We suggest that this SNP is a risk factor for HIV‐infected individuals. *TLR7* identifies guanosine (G) and uridine (U) rich single‐stranded RNA (ssRNA) of HIV.^[^ ^104^ ^]^ The *TLR7* response to HIV is time‐dependent. *TLR7* prevents HIV production and increases antiviral responses.^[^ ^105^ ^]^ *TLR8* also identifies HIV ssRNA.^[^ ^109^ ^]^ *TLR8* induces inflammatory responses that encourage HIV‐1 replication and latency reversal.^[^ ^110^ ^]^

*TLRs* consist of genes associated with mediators of innate and adaptive immunity.^[^ ^18^ ^]^ *TLRs* play a role in recognizing viral particles. Activation of the *TLR* pathways results in the secretion of pro‐inflammatory cytokines. *TLR* is associated with TB. Genetic variations in these genes have shown an increase in TB susceptibility. There were various *TLR2* polymorphisms found in tuberculosis patients. *TLR2* polymorphisms are a risk factor for tuberculosis infection.^[^ ^94^, ^95^, ^96^ ^]^ Yim et al. observed that *TLR‐2* deficiency makes patients more TB‐susceptible.^[^ ^97^ ^]^ We suggest that genetic variations that result in *TLR2* deficiency can be a risk factor for TB. *TLR4* is involved in the induction of MTB immune responses and contributes to the suppression of infection.^[^ ^102^ ^]^ *TLR4* and *TLR8* are associated with TB pathogenesis, this stems from the number of SNPs in *TLR4* and *TLR8* in TB infected individuals compared to healthy individuals. Thada et al. hypothesized that *TLR4* and *TLR8* heterodimer formation regulates MTB immune responses.^[^ ^103^ ^]^ *TLR7* has been suggested to provide MTB host cell immunity.^[^ ^93^, ^105^, ^106^ ^]^ *TLR9* is a pattern recognition receptor that facilitates MTB recognition and controls MTB‐specific T‐cell responses.^[^ ^112^ ^]^ We suggest that polymorphisms that affect the function of these genes will influence the TB outcome.^[^ ^103^ ^]^

*TLR2*, *TLR4*, and *TLR9* are theoretically important in SARS‐CoV‐2 infection. There are beneficial and harmful effects of *TLR* in SARS‐CoV‐2 infection. *TLRs* are potential targets in controlling early infection and in a vaccine against SARS‐CoV‐2.^[^ ^93^ ^]^ *TLR7* is expressed on dendritic cells and monocytes. *TLR7/8* mediates pro‐inflammatory cytokine production.^[^ ^93^ ^]^ *TLR7* has been associated with SARS‐CoV‐2 pathogenesis. *TLR7/8* identify SARS‐CoV‐2.^[^ ^93^ ^]^ *TLR8* is present in the lungs, and *TLR7* and *TLR8* may lead to SARS‐CoV‐1 cytokine storms.^[^ ^106^ ^]^ More research is needed to elucidate the function of these *TLR* genes in COVID‐19 disease.

Similarly, *TLRs* are involved in the innate immune response to malaria. *TLR9* facilitates recognition of the CpG motif in bacterial DNA, *TLR4* mediates the (lipopolysaccharides) LPS of gram‐negative bacteria recognition and *TLR2* helps recognition of a wide range of ligands.^[^ ^92^ ^]^ Sporozoite activates *TLR2* and induces macrophages to release proinflammatory cytokines. *TLR4* was expressed in correlation with the absolute neutrophil count. Differences in *TLR4* gene expression suggested that neutrophil response plays a role in acute malarial infection. *TLR7* and *TLR8* recognize plasmodium‐derived RNA.^[^ ^107^ ^]^ *TLR8* has been associated with severe infected malaria in Mali children.^[^ ^108^ ^]^ *TLR9* (rs5743836) A > G appears to affect malaria progression and susceptibility in specific populations.^[^ ^113^, ^114^, ^115^ ^]^ In Africa, the rs5743836 polymorphism is more common than in Europe. More research is required for *TLR* genes in all infectious diseases.

## 8 Conclusion and Future Recommendations

This review briefly discusses known host genes and gene variations that have been previously associated with HIV, TB, malaria, and COVID‐19 disease progression. We also touch on the prevalence of variation within these genes in certain ethnicities. The gene functionality, the effect of the gene variation, and gene frequency in different ethnicities need to be addressed for all infectious diseases. In summary, this review briefly shows how human genes are necessary for pathogenesis.^[^ ^213^, ^214^ ^]^ Disruption of host genes might slow down disease progression, eliminate the pathogen, or increase the chance of human survival from infections.

As we understand the host genetic impact on HIV, TB, malaria, and COVID‐19, we can unravel their involvement in disease susceptibility, clinical outcomes, co‐infection, and interindividual response. Gene association studies have contributed immensely to drug and therapeutic development for infectious diseases thus far. There are opportunities for future studies to replicate the studies mentioned above to identify host gene targets and do an in‐depth analysis of the effect of host gene variation that is associated with malaria, TB, HIV, and COVID‐19. This is promising for the rapid development of safe and effective therapeutics^[^ ^215^ ^]^ to target the common genes with similar effects on disease. Ultimately, this could be protective against more than one infection. Different populations have different responses to infectious diseases.^[^ ^14^, ^15^, ^16^ ^]^ Therefore, large‐scale studies in different endemic regions, including various ethnic groups are required to better understand the host‐pathogen interaction, pathogenesis, the epidemiological differences between gene expression, the clinical manifestation of infectious disease, why different populations might differ in their susceptibility and severity to HIV, TB, malaria, and SARS‐CoV‐2 infection, and which genes are important drug targets. The host genetics associated with these diseases need to be studied more in an African population. New treatment strategies that are affordable, safe, and effective for Africans need to be developed for infectious diseases. In addition, this study could be carried out for various other diseases common in other populations and this will contribute to precision medicine based on the population's genetics.

## Conflict of Interest

The authors declare no conflict of interest.

## Author Contributions

All authors listed have made a substantial, direct, and intellectual contribution to the work and approved it for publication. All authors have read and agreed to the published version of the manuscript.
